# Safety and efficacy of libevitug in patients with chronic hepatitis B and D virus coinfection: A single-center, open-label, phase IIa trial in China

**DOI:** 10.1097/HC9.0000000000000974

**Published:** 2026-07-15

**Authors:** Xinrui Wang, Xiumei Chi, Yingyu Zhang, Fei Kong, Xin Zhou, Yumei Gu, Yonghe Qi, Liangfeng Zou, Zhongmin Zhou, Jiaying Wen, Pan Chen, Guangyu Li, Yin Zhang, Bin Ye, Bin Chen, Jianhua Sui, Wenhui Li, Hao Zhou, Junqi Niu

**Affiliations:** 1 Department of Hepatology, Center for Pathogen Biology and Infectious Diseases, The First Hospital of Jilin University, Changchun, China; 2 Huahui Health Ltd., Beijing, China; 3 Hygieia Pharmaceuticals Co., Ltd., Shanghai, China; 4 National Institute of Biological Sciences, Beijing, China; 5 Tsinghua Institute of Multidisciplinary Biomedical Research, Tsinghua University, Beijing, China

**Keywords:** anti-PreS1, antiviral, chronic hepatitis D, HBV, HDV, HH-003, human monoclonal antibody

## Abstract

**Background:**

Chronic HBV and HDV coinfection represents the most severe form of viral hepatitis. This study aimed to assess the safety and efficacy of libevitug (HH-003), a novel human monoclonal antibody targeting the PreS1 domain of the HBV large envelope protein, in patients with chronic HBV and HDV coinfection.

**Methods:**

This single-center, open-label, proof-of-concept phase IIa clinical trial enrolled 9 participants who received i.v. infusion of 20 mg/kg libevitug every 2 weeks for 24 weeks, followed by a 24-week follow-up period.

**Results:**

At week 24, 8 participants (88.9%) exhibited a reduction in HDV RNA ≥1 log_10_ from baseline, with 4 (44.4%) achieving undetectable levels. 7 participants (77.8%) achieved virological response (undetectable HDV RNA or ≥2 log_10_ decline from baseline). Among the 5 participants with elevated baseline ALT, 3 (60.0%) achieved a combined virological and biochemical response (ALT normalization). At week 48, 6 participants (66.7%) maintained the virological response achieved at the end of treatment (week 24), with 3 (33.3%) maintaining undetectable HDV RNA levels. Among the 7 participants with quantifiable HBV DNA at baseline, 2 (28.6%) achieved undetectable HBV DNA or ≥1 log_10_ reduction at week 24. Serum HBsAg levels remained unchanged throughout the study period. Treatment-emergent adverse events were reported in 8 participants (88.9%), with only 1 participant (11.1%) experiencing treatment-related adverse events. No grade 3 or serious adverse events were reported, and no discontinuations due to adverse events occurred.

**Conclusions:**

Libevitug demonstrated promising antiviral activity, achieving substantial HDV RNA reduction, a high rate of ALT normalization, and favorable safety and tolerability in participants with chronic HBV and HDV coinfection.

## INTRODUCTION

Chronic hepatitis D is a liver inflammation caused by chronic infection of HDV, a replication-defective RNA virus that requires HBV envelope proteins for hepatocyte entry, particle assembly, and release.^1^ Both HDV and HBV utilize sodium taurocholate cotransporting polypeptide (NTCP) as an entry receptor to infect hepatocytes.^2^ Chronic HDV infection is regarded as the most severe form of viral hepatitis, often leading to more rapid progression to liver cirrhosis, HCC, and liver-related mortality compared with HBV mono-infection.^3^^,^^4^ Chronic HDV infection affects ~12–72 million individuals worldwide.^5^^–^^7^ The off-label use of pegylated interferon-alpha has long been the sole treatment option for patients with chronic hepatitis D.^8^ However, pegylated interferon-alpha therapy only benefits a limited subset of patients and is frequently poorly tolerated, with a high rate of virological relapse following treatment cessation.^9^^,^^10^ The nucleos(t)ide reverse transcriptase inhibitors (NRTIs) exhibit negligible antiviral activity against HDV, as they do not directly inhibit HDV replication.^11^ Currently, bulevirtide is the only therapy approved by the European Medicines Agency (EMA) for the treatment of chronic HDV infection.^12^^–^^14^ It is a linear, 47-amino-acid chemically synthesized lipopeptide that specifically binds to the NTCP receptor.^12^ The optimal dose, treatment duration, and long-term safety of bulevirtide remain unclear.^15^

The persistence of HDV RNA replication in patients with chronic HDV infection is strongly associated with liver disease progression and poor clinical outcome,^16^^–^^18^ highlighting the urgent need for therapeutic strategies to achieve sustained virological suppression. HDV relies on the presence of HBV envelope proteins for hepatocyte entry and replication, specifically utilizing the PreS1 domain of the HBV large envelope protein to mediate HBV/HDV entry via binding to the NTCP receptor.^2^ Blocking the initial entry pathway of HBV and HDV offers a promising therapeutic approach for the treatment of HDV infections. It is clear that viral entry inhibitors can inhibit HBV/HDV entry into hepatocytes and prevent de novo extracellular virus spreading by blocking the interaction between HBV/HDV virions and NTCP receptor. This mechanism is expected to block de novo infection of naive hepatocytes and reinfection of already infected hepatocytes, potentially leading to a progressive reduction in the number of infected hepatocytes and ultimately the clearance of HDV infection as infected hepatocytes turn over. The clinical efficacy of targeting HDV entry mechanisms has been demonstrated by bulevirtide, which achieves significant reductions in HDV RNA viremia and decreased viral burden in hepatocytes among patients with chronic HDV infection following long-term treatment.^13^^,^^14^^,^^19^^–^^21^

Libevitug (HH-003) is a human monoclonal antibody that targets the PreS1 domain of the large envelope protein of HBV. It binds to residues 19–27 of PreS1, a region adjacent to the C-terminus of its receptor binding sites.^22^ By specifically preventing the binding of PreS1 to the NTCP receptor, it effectively blocks viral infection and reinfection of hepatocytes.^22^ Libevitug has shown broad-spectrum neutralization activities against both HBV and HDV infections with higher potency than that of hepatitis B immune globulin in vitro.^22^ In preclinical studies using HBV and HDV infection mouse models, libevitug prevented viral infection, decreased viral titers, and the levels of the small envelope antigen.^22^^–^^24^ In addition, libevitug has demonstrated good safety and favorable tolerability in healthy volunteers (CTR20182292) and in patients with chronic HBV infection (NCT05542979). In this proof-of-concept study, we assessed the safety and efficacy of libevitug over a 24-week treatment period and a 24-week follow-up in participants with chronic HBV and HDV coinfection.

## METHODS

### Study design and participants

The HH003-201 study was designed as a single-center, single-arm, open-label phase IIa trial, and it was conducted at The First Hospital of Jilin University (Jilin, China) from August 2021 to January 2023. The study protocol and informed consent forms were reviewed and approved by the Ethics Committee of the First Hospital of Jilin University Clinical Research Institute (Approval Number 21Y139-001). The study was conducted in accordance with the International Conference on Harmonization Good Clinical Practice Guideline and the Declaration of Helsinki. All participants received comprehensive written and oral information regarding the study, and written informed consent was obtained before the screening for eligibility. The study was registered at clinicaltrials.gov under the identifier NCT05674448.

Male or female participants (aged 18–70 y) with a body mass index of 18–32 kg/m^2^ were eligible for enrollment if they were serum anti-HDV IgG and HDV RNA positive and had a history of HBV infection for at least 6 months at screening. Participants with decompensated liver cirrhosis or liver dysfunction (including but not limited to ascites, HE, and upper gastrointestinal bleeding) within 3 months before screening or a history of HCC were excluded. Other key exclusion criteria included a serum total bilirubin >1.5× upper limit of normal (ULN); DBil >1.5× ULN; ALT> 5× ULN; albumin <35 g/L; coinfection with HIV or syphilis; and interferon antiviral therapy within 1 year before screening. The full eligibility criteria are provided in Supplemental Material, http://links.lww.com/HC9/C360.

### Procedure

All 9 eligible participants received 20 mg/kg libevitug intravenously every 2 weeks for 24 weeks, followed by a 24-week follow-up period. In this study, 1 participant with prior entecavir (ETV) anti-HBV treatment continued ETV (oral, 0.5 mg, once daily) as background therapy throughout the study period. Of the remaining 8 participants who did not receive NRTIs during the 24-week treatment period, 6 initiated ETV treatment following the end-of-treatment visit, as determined by the investigator, while the remaining 2 participants did not receive NRTIs. Due to the COVID-19 pandemic, 6 participants missed a single dose of libevitug during the treatment period, while 3 participants missed 1 follow-up visit during the follow-up period.

Safety was assessed by routine clinical examinations including vital signs monitoring and various laboratory tests, such as hematology, biochemistry, coagulation, and liver function tests. Electrocardiography and symptom-directed physical examinations were also performed. All adverse events (AEs) and laboratory abnormalities were graded according to the Common Terminology Criteria for Adverse Events (CTCAE) version 5.0.

The efficacy of libevitug treatment was assessed by monitoring changes in liver function tests and virological HDV and HBV biomarkers. Serum ALT levels were quantified using the Roche Cobas 6000/c501 system (Alanine aminotransferase acc. to IFCC R1 and R2, Roche Diagnostics). The ULN for serum ALT was established at 33 U/L for women and 41 U/L for men according to the central laboratory criteria. ALT flare was defined as a >3-fold increase in serum ALT level above baseline. Serum HDV RNA was quantified using the RoboGene HDV RNA quantification kit 2.0 (Roboscreen GmbH), subsequent to automated magnetic-bead nucleic acid extraction (GENEWAY.bio), with a limit of detection (LOD) of 6 IU/mL. Plasma HBV DNA was quantified using the Cobas HBV Test (Roche Diagnostics), which has a lower limit of quantification (LLOQ) of 10 IU/mL. Serum HBsAg levels were measured using the Elecsys HBsAg II quantitative assay (Roche Diagnostics GmbH), with an LLOQ of 0.05 IU/mL, while HBcrAg levels were measured using the LUMIPULSE G HBcrAg assay (Fujirebio Europe), with an LLOQ of 3 log_10_ U/mL.^25^ Serum HBV pgRNA was quantified using a quantitative reverse transcription-qPCR with an LLOQ of 1000 copies/mL.^26^

In addition, HBV and HDV genotypes were determined by sequence analysis based on Nested-PCR as described.^27^^,^^28^ For samples in which HBV genotype could not be determined by sequencing, an enzyme immunoassay–based HBV genotyping kit (Cosmo Bio) was used.

To assess possible viral resistance to libevitug, the potential presence of amino acid substitutions in the HBV PreS1 domain, the HBV reverse transcription region, and the hepatitis delta antigen region was examined at baseline and at the study visit when virological breakthrough occurred, using Sanger sequencing. Virological breakthrough was defined as a confirmed increase in HBV DNA or HDV RNA levels of >1 log_10_ from the nadir in patients with prior reduction of at least >1 log_10_ reduction from baseline, or as HBV DNA level becoming detectable in patients who had previously achieved undetectable levels, or re-detectable HDV RNA at levels 10-fold above the lower LOD after HDV RNA had become undetectable. The detection methods are detailed in Supplemental Material, http://links.lww.com/HC9/C360.

### Efficacy and safety endpoints

The primary endpoints were the proportion of participants achieving (1) undetectable serum HDV RNA or ≥1 log_10_ decline from baseline (undetectable HDV RNA defined as target not detected, TND), (2) undetectable plasma HBV DNA or ≥1 log_10_ reduction compared with baseline (undetectable HBV DNA defined as HBV DNA<LLOQ, 10 IU/mL), and (3) the reduction in serum HBsAg levels from baseline at week 24. Secondary endpoints included the proportion of participants with abnormal baseline ALT levels who achieved ALT normalization at week 24; changes in serum HBsAg, plasma HBV DNA, and serum HDV RNA levels from baseline at visit time points during treatment and up to 24 weeks after treatment. In addition, virological response (the proportion of participants with undetectable HDV RNA or ≥2 log_10_ decline from baseline), biochemical response (the proportion of participants who achieved normal ALT levels), and combined response (both virological and biochemical response) were assessed at week 24 and week 48 post hoc.

The safety endpoints included AEs, physical examination findings, vital signs, 12­-lead ECG, abdominal ultrasound scans, liver stiffness measurement, and abnormal laboratory results.

### Statistical analysis

No formal sample size calculation was performed for this exploratory study. Nine participants were deemed sufficient to provide preliminary results on the safety and efficacy of libevitug in the current participant population. All enrolled participants were included in the analysis for all assessed parameters. Antiviral activity was summarized based on the observed data, with missing data being excluded from the summaries. Categorical variables are presented as frequencies (percentages), while continuous variables are expressed as medians (range) or means (SD). Graphs and statistical analysis were performed using SAS (version 9.4), Microsoft Excel, or R (version 4.0). For outcome statistical analyses, HBV DNA, HBsAg, HBV pgRNA, and HBcrAg samples below the assay lower limit of quantitation were assigned an LLOQ value; HDV RNA samples below the assay LOD were assigned an LOD value.

## RESULTS

### Participant demographics and characteristics

Between August 2021 and June 2022, a total of 19 participants with chronic HBV and HDV coinfection were screened. Of these, 9 participants fulfilled the inclusion and exclusion criteria and were enrolled to receive an i.v. infusion of 20 mg/kg of libevitug every 2 weeks. All 9 participants completed the 24-week treatment and the subsequent 24-week follow-up period (Figure 1). Six participants missed a single dose of libevitug owing to COVID-19. Of the 10 individuals not enrolled, 4 were HBsAg-negative, 2 had HCC, 1 was HDV RNA-negative, 1 was HDV IgG negative with poorly controlled hypertension, 1 was deemed unsuitable by the investigator due to severe conditions, and 1 had decompensated cirrhosis.

**FIGURE 1:**
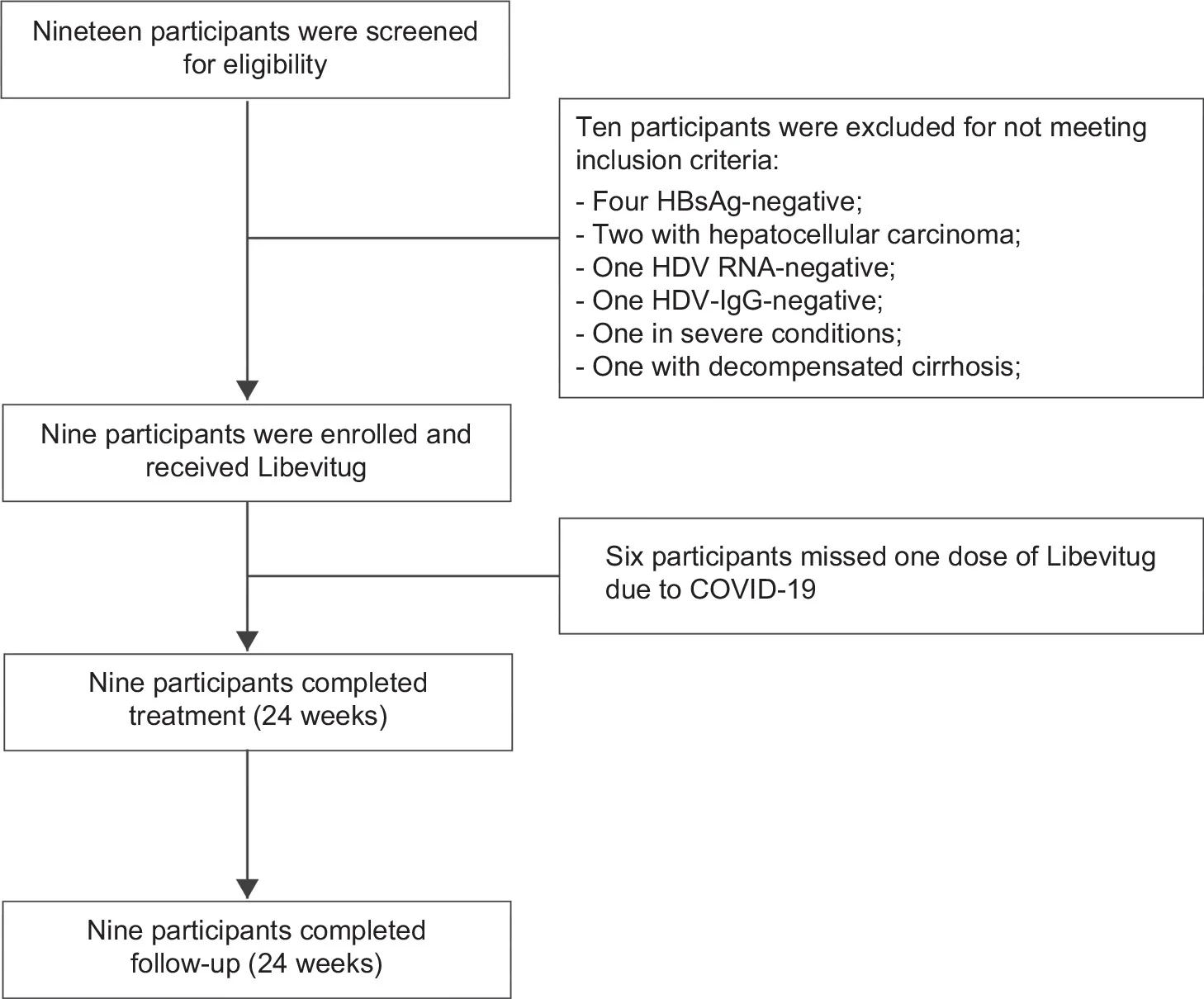
Trial profile.

The baseline demographic and clinical characteristics are shown in Table 1. Overall, the median age of the participants was 57 years (49, 65), 6 (66.7%) were male and 3 (33.3%) were female. All participants were HBeAg-negative and exhibited HBV pgRNA levels below the LLOQ at screening. No participant had compensated cirrhosis. Three enrolled participants (33.3%) had a history of HCV infection but were negative for HCV-Ag at screening. One participant (11.1%) was receiving ETV (oral, 0.5 mg, once daily) before the screening and continued this regimen throughout the study. The median body mass index was 23.7 kg/m^2^ (19.3, 28.2). Median liver stiffness measurements, platelet counts, and APRI score were 8.9 kPa (3.6, 14.1), 195.0×10^9^/L (153.0, 223.0), and 0.5 (0.4, 1.7), respectively. The mean (SD) ALT level was 46.2±22.7 U/L, with 5 participants (55.6%) exhibiting elevated baseline ALT levels (>33 U/L for women and >41 U/L for men). The mean (SD) HDV RNA, HBV DNA, HBsAg, and HBcrAg levels were 3.6±1.2 log_10_ IU/mL, 2.2±1.2 log_10_ IU/mL, 3.2±0.6 log_10_ IU/mL, and 3.5±0.5 log_10_ U/mL, respectively. Eight out of 9 participants (88.9%) were infected with HDV genotype 1, whereas the HDV genotype for 1 participant could not be determined because the HDV RNA level was below the detection limit of the assay. HBV genotypes B, C, and D were equally distributed, with 3 participants (33.3%) for each HBV genotype (Supplemental Figure S1, http://links.lww.com/HC9/C360).

**TABLE 1:** Participant demographics and clinical characteristics

Baseline characteristics	Libevitug (N=9)
Age, y	
Median (min, max)	57 (49, 65)
Sex, n (%)	
Male	6 (66.7)
Female	3 (33.3)
Race, n (%)	
Asian	9 (100.0)
BMI, kg/m^2^	
Median (min, max)	23.7 (19.3, 28.2)
ALT, U/L	
Median (min, max)	36.0 (21.0, 86.0)
Mean±SD	46.2±22.7
>ULN, n (%)	5 (55.6)
LSM, kPa	
Median (min, max)	8.9 (3.6, 14.1)
Mean±SD	9.0±3.6
Compensated cirrhosis, n (%)^a^	0 (0.0)
HBeAg positive status, n (%)	0 (0.0)
Anti-HBe positive status, n (%)	9 (100.0)
HDV genotype, n (%)	
Genotype 1	8 (88.9)
Not detected	1 (11.1)
HBV genotype, n (%)	
Genotype A	0 (0.0)
Genotype B	3 (33.3)
Genotype C	3 (33.3)
Genotype D	3 (33.3)
HDV RNA, log_10_ IU/mL	
Median (min, max)	3.5 (1.6, 5.4)
Mean±SD	3.6±1.2
HBsAg, log_10_ IU/mL	
Median (min, max)	3.3 (1.7, 3.7)
Mean±SD	3.2±0.6
HBV DNA, log_10_ IU/mL	
Median (min, max)	1.7 (1.0, 4.2)
Mean±SD	2.2±1.2
<10 IU/mL, n (%)	2 (22.2)
HBV pgRNA, n (%)	
<LLOQ, 3.0 log_10_ copies/mL	9 (100.0)
HBcrAg, log_10_ U/mL	
Median (min, max)	3.4 (3.0, 4.6)
Mean±SD	3.5±0.5
>3.0 log_10_ U/mL, n (%)	7 (77.8)
Ongoing NRTIs treatment, n (%)	1 (11.1)
Platelet count (×10^9^/L)	
Median (min, max)	195.0 (153.0, 223.0)
Mean±SD	195.8±23.6
APRI score	
Median (min, max)	0.5 (0.4, 1.7)
Mean±SD	0.7±0.4

^a^ Cirrhosis was assessed using abdominal ultrasound combined with liver stiffness measurements via FibroScan.

Abbreviations: APRI, aspartate aminotransferase-to-platelet ratio index; BMI, body mass index; HBcrAg, hepatitis B core-related antigen; LLOQ, lower limit of quantification; LSM, Liver stiffness measurement; NRTIs, nucleos(t)ide reverse transcriptase inhibitors; pgRNA, pregenomic RNA; ULN, upper limit of normal.

### Efficacy

#### HDV RNA

The mean (SD) HDV RNA levels at baseline were 3.6 (1.2) log_10_ IU/mL. After libevitug treatment, the mean (SD) reductions of HDV RNA level from baseline at week 24 and week 48 were 2.1 (1.1) log_10_ and 1.6 (1.4) log_10_, respectively. By the end of treatment (week 24), 8 participants (88.9%) achieved a ≥1 log_10_ reduction in HDV RNA from baseline, with 4 participants (44.4%) achieving undetectable HDV RNA levels. Notably, HDV RNA levels of 3 participants (33.3%) remained undetectable at the end of the study (week 48). Individual changes in HDV RNA levels over time are shown in Figure 2A.

**FIGURE 2:**
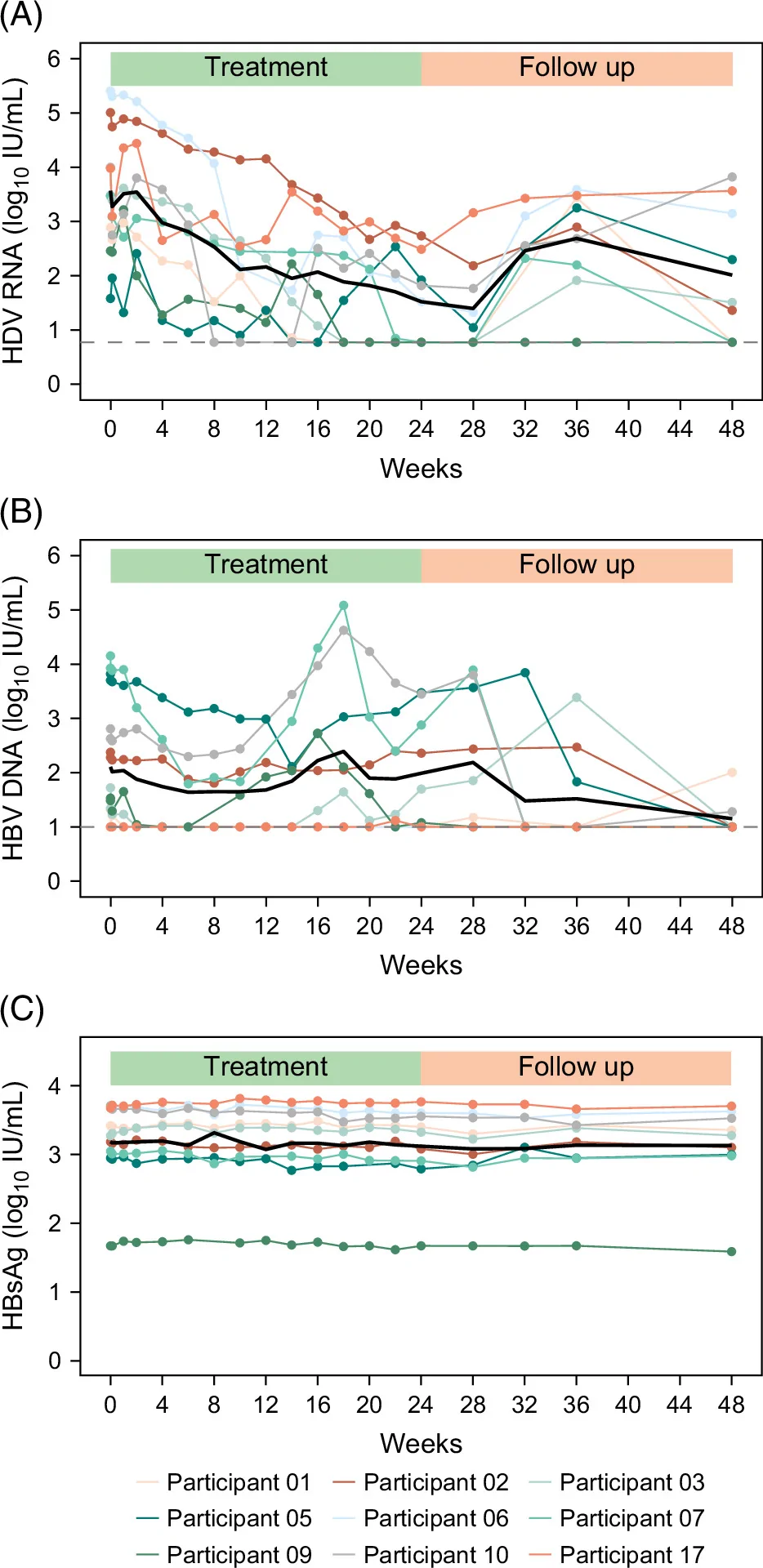
Changes in HDV RNA, HBV DNA, and HBsAg during treatment and follow-up period. Individual, color-coded participant responses during the 24-week treatment and the 24-week follow-up are shown for serum HDV RNA (A), plasma HBV DNA (B), and serum HBsAg (C). Thick black lines indicate mean values and dashed gray lines indicate the LOD (6 IU/mL) for HDV RNA and the LLOQ (10 IU/mL) for HBV DNA. Due to missed visits and (or) dosing, missing data occurred for the following participants: participant 01 (week 32), participant 02 (week 32), participant 03 (week 32), participant 05 (week 20), participant 06 (week 12), participant 07 (week 12), participant 09 (week 8), participant 10 (week 12), and participant 17 (week 6). Abbreviations: LLOQ, lower limit of quantification; LOD, limit of detection.

#### HBV DNA

Among 7 participants with detectable HBV DNA (>10 IU/mL) at baseline, 2 participants (28.6%) achieved undetectable HBV DNA or ≥1 log_10_ reduction from baseline by week 24. Notably, 2 participants experienced rapid and profound HBV DNA decreases (maximum reduction of 1.7 log_10_ and 2.3 log_10_ from baseline) before missing 1 dose of libevitug due to COVID-19. During the study period, HBV DNA fluctuated, partly attributable to 6 participants missing a dose of libevitug during the 24-week treatment period. In addition, 6 participants initiated NRTI treatment during follow-up. The mean (SD) HBV DNA decrease from baseline was 0.2 (0.5) log_10_ at week 24 and 1.0 (1.3) log_10_ at week 48. By week 48, 7 participants achieved HBV DNA levels below LLOQ. Individual changes in HBV DNA levels over time are shown in Figure 2B.

#### HBV pgRNA, HBsAg, and HBcrAg

All 9 participants demonstrated undetectable HBV pgRNA at baseline and remained below the LLOQ (1000 copies/mL) throughout the study. No clinically significant changes in serum HBsAg levels were observed, and none of the 9 participants achieved HBsAg loss or seroconversion. Individual changes in HBsAg levels over time are shown in Figure 2C. For HBcrAg, at baseline, 2 participants showed undetectable serum HBcrAg (<LLOQ, 3.0 log_10_ U/mL). Among the 7 participants who had baseline HBcrAg levels above the LLOQ, 3 achieved HBcrAg loss (<LLOQ, 3.0 log_10_ U/mL), while the remaining 4 participants showed no changes in HBcrAg levels from baseline by week 24. At week 48, most participants showed no substantial changes in HBcrAg levels from the baseline (Supplemental Figure S2, http://links.lww.com/HC9/C360).

#### ALT

Among the 5 participants who had abnormal baseline ALT levels, 3 of 5 (60.0%) participants achieved ALT normalization at week 24, and 2 of the 5 (40.0%) achieved ALT normalization at week 48. The other 4 participants remained within the normal range of ALT levels at week 24 and 3 (75.0%) maintained normal ALT levels at week 48, while 1 participant experienced an ALT elevation. The individual changes in ALT levels over time are shown in Figure 3.

**FIGURE 3:**
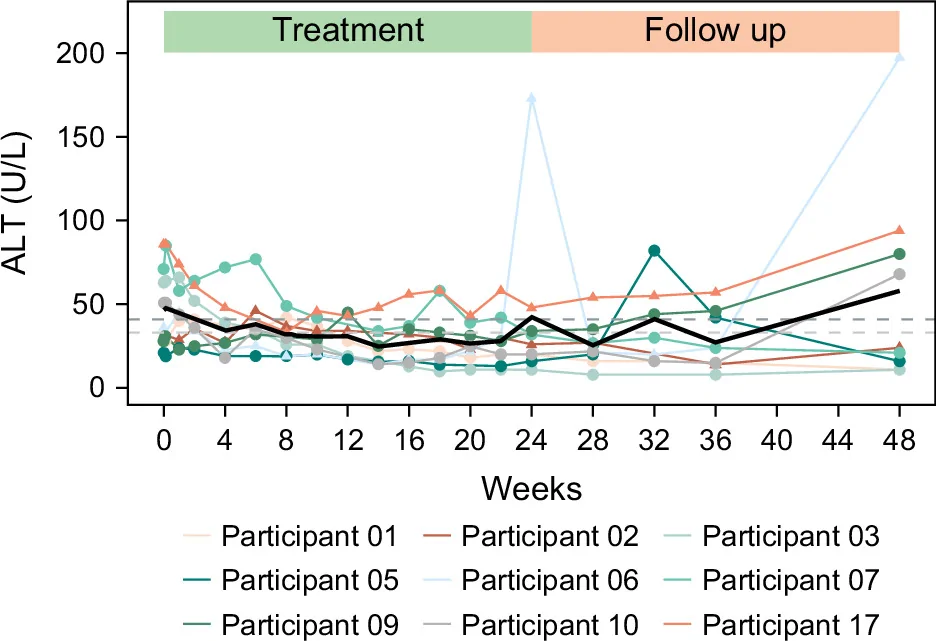
Change in ALT during treatment and follow-up period. Individual, color-coded participant responses in ALT during the 24-week treatment and the 24-week follow-up. Thick black lines indicate mean values, and dashed lines indicate the ULN of ALT for male (41 U/L, blue) and female (33 U/L, orange). Circles represent male, and triangles represent female. Due to missed visits and (or) dosing, missing data occurred for the following participants: participant 01 (week 32), participant 02 (week 32), participant 03 (week 32), participant 05 (week 20), participant 06 (week 12), participant 07 (week 12), participant 09 (week 8), participant 10 (week 12), and participant17 (week 6). Abbreviation: ULN, upper limit of normal.

Furthermore, virological response, biochemical response, and combined response were assessed at week 24 and week 48 post hoc. At week 24, 7 of the 9 participants (77.8%) achieved virological response. Among the 5 participants with abnormal baseline ALT levels, 3 (60.0%) achieved biochemical response and combined response. At week 48, 6 of 9 (66.7%) and 2 of 5 (40.0%) participants achieved virological response and combined response, respectively (Figure 4).

**FIGURE 4:**
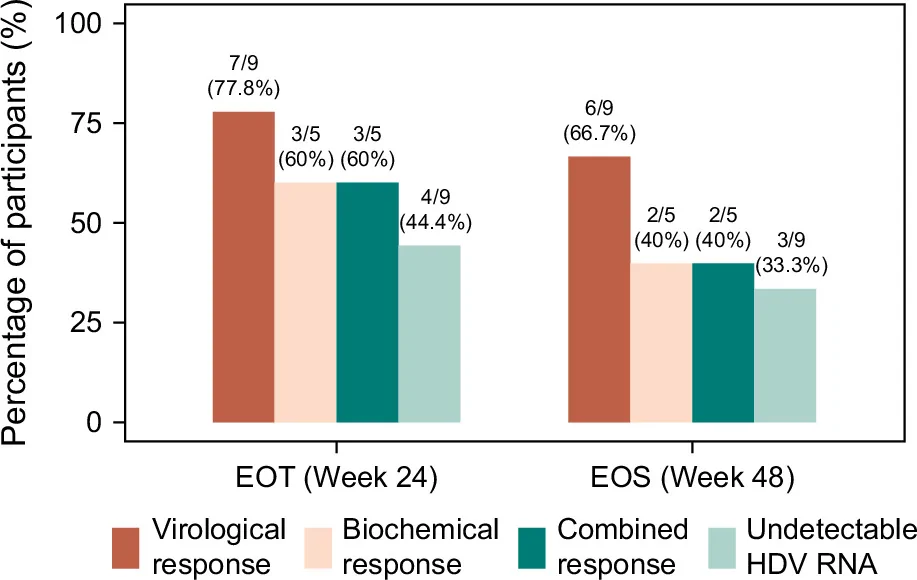
Virological response, biochemical response, and combined response at weeks 24 and 48. Virological response was defined as the proportion of participants with undetectable HDV RNA or ≥2 log_10_ decline from baseline. Biochemical response was defined as the proportion of participants who achieved ALT normalization. Combined response was defined as the proportion of participants who achieved both virological and biochemical responses. Undetectable HDV RNA was defined as TND. Abbreviations: EOS, end of study; EOT, end of treatment; TND, target not detected.

### Adverse events

Overall, treatment-emergent adverse events (TEAEs) were reported in 8 participants (88.9%). All TEAEs were mild in severity, being of grade 1 or 2 according to the Common Terminology Criteria for Adverse Events (CTCAE) version 5.0. No grade 3 or higher TEAEs, serious TEAEs, or TEAEs leading to death were observed. In addition, no TEAEs necessitated study treatment withdrawal or discontinuation.

Among the 8 participants, only 1 participant (11.1%) experienced treatment-related AEs, including abdominal discomfort (grade 1) and asthenia (grade 2). Both AEs were transient and resolved without requiring additional intervention. The TEAEs are summarized in Table 2. Monitoring laboratory variables, including serum ALT, AST, ALP, and GGT, showed a general decline relative to baseline. The mean bile acid levels were slightly elevated.

**TABLE 2:** Summary of TEAEs

	Libevitug (20 mg/kg, Q2W, N=9)			
	Grade 1 (n, %)	Grade 2 (n, %)	≥Grade 3 (n, %)	Total (n, %)
Any TEAEs	7 (77.8)	1 (11.1)	0	8 (88.9)
Metabolism and nutrition disorders				
Hypertriglyceridemia	3 (33.3)	0	0	3 (33.3)
Hypercholesterolemia	2 (22.2)	0	0	2 (22.2)
Hyperuricemia	2 (22.2)	0	0	2 (22.2)
Hypo HDL cholesterolemia	1 (11.1)	0	0	1 (11.1)
Renal and urinary disorders				
Hematuria	3 (33.3)	0	0	3 (33.3)
Nephrolithiasis	0	1 (11.1)	0	1 (11.1)
Renal cyst	1 (11.1)	0	0	1 (11.1)
Hepatobiliary disorders				
Cholecystitis	1 (11.1)	0	0	1 (11.1)
Cholelithiasis	1 (11.1)	0	0	1 (11.1)
Hepatic cirrhosis	1 (11.1)	0	0	1 (11.1)
Hepatic cyst	1 (11.1)	0	0	1 (11.1)
Hepatic mass	1 (11.1)	0	0	1 (11.1)
Hepatic steatosis	1 (11.1)	0	0	1 (11.1)
Gastrointestinal disorders				
Abdominal discomfort	1 (11.1)	0	0	1 (11.1)
Abdominal distension	1 (11.1)	0	0	1 (11.1)
Infections and infestations				
Urinary tract infection	2 (22.2)	0	0	2 (22.2)
Various examinations				
Blood glucose increased	2 (22.2)	0	0	2 (22.2)
General disorders and administration site conditions				
Asthenia	0	1 (11.1)	0	1 (11.1)

Adverse events were coded according to the Medical Dictionary for Regulatory Activities (MedDRA) version 25.1.

Abbreviations: Q2W, once every 2 weeks; TEAE, treatment-emergent adverse event.

### Drug resistance

Five participants met the protocol-defined virological breakthrough criteria during the trial. Of these, 3 participants (33.3%) experienced HBV DNA virological breakthrough while maintaining undetectable HDV RNA levels at week 24; 2 participants (22.2%) demonstrated concurrent HBV and HDV virological breakthrough; and no participants experienced HDV-only virological breakthrough. Notably, 4 of the 5 participants (80.0%) experienced virological breakthrough at the study visit after missing a dose of libevitug treatment due to COVID-19. After resuming libevitug, all 4 participants showed significant decreases (>0.5 log_10_ decline from the maximum viral load) in HBV DNA and/or HDV RNA levels by week 24.

Sequencing analyses were conducted on samples obtained from the five participants at baseline and at the visit time point of virological breakthrough occurrence. No treatment-emergent amino acid substitutions were detected in the reverse transcriptase region of the HBV polymerase gene or HDV delta antigen across all 5 participants. Amino acid substitutions in the PreS1 domain of the HBV large envelope protein were found in 4 of the 5 participants, specifically A22T, A22T/A, F23L/F, and A22D/G/A (Supplemental Table S2, http://links.lww.com/HC9/C360). No amino acid substitutions in the HBV PreS1 domain were detected in any of the remaining participants. Despite the presence of these amino acid substitutions, 3 of the 4 participants achieved undetectable HDV RNA, and 1 had a 2.2 log_10_ reduction in HDV RNA by week 24. In addition, 3 of the 4 participants showed HBV DNA declines of 2.2 log_10_, 1.6 log_10_, and 1.2 log_10_, respectively, from the peak viral loads. These data suggest that the identified amino acid substitutions in the PreS1 domain had minimal or limited impact on the antiviral activity of libevitug.

## DISCUSSION

This study demonstrated that the 24-week treatment with libevitug resulted in a significant proportion of participants with chronic HBV and HDV coinfection achieving virological response, biochemical response, and combined response. These markers are considered meaningful predictors of improved clinical outcomes in patients with hepatitis D.^29^^,^^30^

The large (L) envelope protein of HBV contains an PreS1 domain at the N-terminal of S domain and plays a pivotal role in HBV and HDV infection.^31^^,^^32^ The PreS1 domain specifically binds to NTCP receptor in hepatocytes and mediates viral entry of HBV and HDV.^2^ Libevitug, a therapeutic antibody against the PreS1 domain, inhibits HBV and HDV virions binding to NTCP by targeting a highly conserved epitope adjacent to the PreS1-receptor binding site.^2^^,^^22^ Preclinical studies have previously demonstrated that libevitug exhibits remarkable prophylactic efficacy against de novo HDV and HBV infections in murine models and shows significant therapeutic potential for HBV infection, with Fc-dependent effector functions contributing to its anti-HBV activity in vivo.^22^^–^^24^

In this proof-of-concept study, all participants with chronic HBV and HDV coinfection under libevitug treatment experienced a decline in HDV RNA. These efficacy findings are consistent with previously reported clinical data on bulevirtide,^33^ supporting their similar mechanisms of action as entry blockers. Both inhibit viral entry by blocking the interaction between HBV/HDV virions and the NTCP receptor, leading to a sustained decline in HDV RNA viremia. In this study, which enrolled participants with a median baseline HDV RNA of 3.48 (1.58–5.40) log_10_ IU/mL, libevitug monotherapy demonstrated a rapid decline in HDV RNA levels, with a mean reduction of 2.07 log_10_ and a high rate of sustained virologic suppression, with 44.4% (4 of 9) of participants achieving undetectable HDV RNA at week 24. Notably, 33.3% (3 of 9) maintained undetectable HDV RNA after the 24-week follow-up, suggesting potential durability of the virological response. The mean baseline levels of HDV RNA and ALT of patients in this study were lower than those reported in some other studies.^34^ The differences may be due to demographic factors, including age, associated with the small sample size of the trial. In the MYR201 trial (with a total of 7 participants), bulevirtide (Myrcludex B, 2 mg, daily, s.c.) monotherapy resulted in a mean reduction of ~1.67 log_10_ in HDV RNA by week 24.^35^ However, both trials were small, proof-of-concept studies with relatively short treatment durations and different baseline patient characteristics. In the MYR301 phase III trial (N=150), after 144 weeks of bulevirtide monotherapy, a combined response was achieved in 57% and 54% of participants with chronic hepatitis D receiving 2 and 10 mg, respectively, while virological responses were achieved by 60% and 76% of participants, respectively. Notably, 43% of the nonresponders and 82% of the partial responders identified at the week 24 evaluation achieved a virologic response by week 96,^13^^,^^36^ indicating that prolonged treatment of the entry inhibitor progressively enhances both combined and virological responses.

Serum ALT is a clinically important indicator reflecting hepatic inflammation, hepatocellular injury, and the progression of liver diseases associated with viral hepatitis. ALT normalization is a widely accepted surrogate biomarker for successful treatment response in chronic viral hepatitis.^37^^,^^38^ Among the 5 participants with abnormal baseline ALT levels in this study, 4 exhibited clinically meaningful reductions in ALT levels, with 3 achieving ALT normalization by week 24 and 2 maintaining normal ALT levels through week 48. In addition, 4 participants with normal baseline ALT levels maintained normal ALT levels at week 24, while 3 continued to have normal ALT levels at week 48 (Supplemental Figure S3, http://links.lww.com/HC9/C360). These observations suggest that 24 weeks of libevitug treatment leads to meaningful improvement in liver function in participants with chronic HBV and HDV coinfection.

One female participant who was under ETV treatment and was HBeAg-negative with undetectable HBV pgRNA (<LLOQ, 1000 copies/mL) and suppressed HBV DNA levels (<10 IU/mL), exhibited mild elevation of ALT (36 U/L) at baseline with a serum HDV RNA level of 5.4 log_10_ IU/mL. During libevitug treatment, this participant experienced a gradual normalization of ALT levels, accompanied by substantial reductions in HDV RNA and HBcrAg levels. At week 24, a transient ALT flare (173 U/L) occurred in the participant concomitantly with a 3.9-log_10_ reduction in HDV RNA levels (Supplemental Figure S4A, http://links.lww.com/HC9/C360), with no abnormalities in serum bilirubin levels. The transient ALT elevation likely reflects immune-mediated clearance of HDV-infected hepatocytes. The levels of ALT in this participant normalized during follow-up. However, at week 48, an ALT flare (197 U/L) occurred in the participant coinciding with HDV RNA rebound, indicating HDV replication–induced hepatic inflammation or injury. Another participant experienced ALT flare (82 U/L) at week 32, and the flare was accompanied by increased HDV RNA and HBV DNA levels after cessation of libevitug treatment. Following the administration of NRTI, the HBV DNA level dropped below the LLOQ, and the ALT level returned to normal, indicating HBV replication–induced ALT flare in this participant (Supplemental Figure S4B, http://links.lww.com/HC9/C360).

We also monitored the anti-HBV activity of libevitug by analysis of HBV biomarkers (HBV DNA, HBsAg, HBeAg, HBV pgRNA, and HBcrAg). It is noteworthy that all enrolled participants in this study were HBeAg-negative, with undetectable HBV pgRNA at baseline, while most participants had quantifiable HBcrAg and low-level HBV DNA viremia (<1000 IU/mL). This virological profile aligns with the known suppressive effect of HDV on HBV replication, characterized by reduced HBV transcriptional activity and diminished viral replication markers despite ongoing liver disease activity.^39^^–^^42^ During the early phase of libevitug treatment, all 7 participants with detectable baseline HBV DNA levels exhibited reductions in HBV DNA levels. The maximum decline was observed at week 6, with a mean decrease of 0.7 log_10_ compared with baseline; 2 of the 7 participants (28.6%) achieved undetectable HBV DNA or ≥1 log_10_ reduction from baseline by week 24. Notably, 2 participants experienced rapid and profound HBV DNA decreases (maximum reduction of 1.7 log_10_ and 2.3 log_10_ from baseline). Between weeks 6 and 24, minor fluctuations in HBV DNA were noted, likely attributable to missed doses in 6 participants due to COVID-19. Together, these data demonstrate that libevitug monotherapy exerts clinically meaningful antiviral activity against HBV replication.

Despite the potent, direct antiviral activity of libevitug, as evidenced by HDV RNA suppression and HBV DNA reduction in the participants treated with libevitug, HBsAg levels remained largely stable across all participants, in line with the results observed in the studies of bulevirtide monotherapy.^19^^,^^35^ The result is not unexpected, as it is known that in HBeAg-negative patients, the HBsAg largely originates from hepatocytes harboring integrated HBV DNA rather than cccDNA.^43^^,^^44^ The observed reductions in serum viral load are most likely attributable to a decreased number of infected hepatocytes in the liver, resulting from the inhibition of viral entry.^20^^,^^45^^,^^46^

Libevitug exhibited a favorable safety profile, with treatment-related AEs reported in only 1 of 9 participants, all of which were mild to moderate. No grade 3 or serious AEs were observed, and no treatment discontinuations due to AEs. Unlike bulevirtide, which binds to NTCP and induces a significant increase in serum bile acid levels,^13^^,^^14^^,^^19^^,^^35^^,^^47^ libevitug demonstrated minimal effects on bile acid levels in this study. Although current clinical data indicate that these bile acid elevations induced by bulevirtide have not led to clinically significant AEs or symptomatic safety concerns in treated patients, the long-term consequences of sustained bile acid elevation remain uncertain and require continued monitoring.^33^

We acknowledge that this study has some limitations. First, the small sample size and single-center design potentially limit the generalizability of our findings. Second, the 24-week treatment duration is relatively short to achieve complete suppression of HDV replication, as shown by the rebound of HDV RNA in some participants following treatment discontinuation. This highlights the need for longer treatment durations to better evaluate the long-term efficacy and durability of viral suppression. Finally, this study investigated only a single dose level of 20 mg/kg, and the optimal dosing regimen remains to be further determined.

In conclusion, libevitug represents a promising novel therapeutic option for chronic HBV and HDV coinfection, demonstrating a robust reduction in HDV RNA levels and effective ALT normalization, and a favorable safety profile. The efficacy and safety of 48 weeks of treatment with libevitug is currently being rigorously evaluated in a multicenter, randomized, controlled, open-label, phase IIb study involving ~90 participants with chronic HDV infection (NCT05861674). This study is expected to provide definitive data on the efficacy and safety of libevitug, as well as to clarify the optimal dosing strategy for the treatment of chronic HDV infection.

## Supplementary Material

## References

[1] Urban S Neumann-Haefelin C Lampertico P . Hepatitis D virus in 2021: Virology, immunology and new treatment approaches for a difficult-to-treat disease. Gut. 2021;70:1782–1794. 10.1136/gutjnl-2020-323888 PMC8355886 34103404

[2] Yan H Zhong G Xu G He W Jing Z Gao Z . Sodium taurocholate cotransporting polypeptide is a functional receptor for human hepatitis B and D virus. Elife. 2012;1:e00049. 10.7554/eLife.00049 PMC3485615 23150796

[3] Lampertico P Degasperi E Sandmann L Wedemeyer H Delta Cure Working G . Hepatitis D virus infection: Pathophysiology, epidemiology and treatment. Report from the first international delta cure meeting 2022. JHEP Rep. 2023;5:100818. 10.1016/j.jhepr.2023.100818 PMC10428117 37593170

[4] Wranke A Heidrich B Deterding K Hupa-Breier KL Kirschner J Bremer B . Clinical long-term outcome of hepatitis D compared to hepatitis B monoinfection. Hepatol Int. 2023;17:1359–1367. 10.1007/s12072-023-10575-0 PMC10661878 37789170

[5] Chen HY Shen DT Ji DZ Han PC Zhang WM Ma JF . Prevalence and burden of hepatitis D virus infection in the global population: A systematic review and meta-analysis. Gut. 2019;68:512–521. 10.1136/gutjnl-2018-316601 30228220

[6] Miao Z Zhang S Ou X Li S Ma Z Wang W . Estimating the global prevalence, disease progression, and clinical outcome of hepatitis delta virus infection. J Infect Dis. 2020;221:1677–1687. 10.1093/infdis/jiz633 PMC7184909 31778167

[7] Stockdale AJ Kreuels B Henrion MYR Giorgi E Kyomuhangi I de Martel C . The global prevalence of hepatitis D virus infection: Systematic review and meta-analysis. J Hepatol. 2020;73:523–532. 10.1016/j.jhep.2020.04.008 PMC7438974 32335166

[8] Erhardt A Gerlich W Starke C Wend U Donner A Sagir A . Treatment of chronic hepatitis delta with pegylated interferon-alpha2b. Liver Int. 2006;26:805–810. 10.1111/j.1478-3231.2006.01279.x 16911462

[9] Wedemeyer H Yurdaydin C Hardtke S Caruntu FA Curescu MG Yalcin K . Peginterferon alfa-2a plus tenofovir disoproxil fumarate for hepatitis D (HIDIT-II): A randomised, placebo controlled, phase 2 trial. Lancet Infect Dis. 2019;19:275–286. 10.1016/S1473-3099(18)30663-7 30833068

[10] Sandmann L Wedemeyer H . Interferon-based treatment of chronic hepatitis D. Liver Int. 2023;43(suppl 1):69–79. 10.1111/liv.15410 36002390

[11] Wedemeyer H Yurdaydìn C Dalekos GN Erhardt A Çakaloğlu Y Değertekin H . Peginterferon plus adefovir versus either drug alone for hepatitis delta. N Engl J Med. 2011;364:322–331. 10.1056/NEJMoa0912696 21268724

[12] Kang C Syed YY . Bulevirtide: First approval. Drugs. 2020;80:1601–1605. 10.1007/s40265-020-01400-1 32926353

[13] Wedemeyer H Aleman S Brunetto M Blank A Andreone P Bogomolov P . Bulevirtide monotherapy in patients with chronic HDV: Efficacy and safety results through week 96 from a phase III randomized trial. J Hepatol. 2024;81:621–629. 10.1016/j.jhep.2024.05.001 38734383

[14] Asselah T Chulanov V Lampertico P Wedemeyer H Streinu-Cercel A Pântea V . Bulevirtide combined with pegylated interferon for chronic hepatitis D. N Engl J Med. 2024;391:133–143. 10.1056/NEJMoa2314134 38842520

[15] EASL . EASL Clinical Practice Guidelines on hepatitis delta virus. J Hepatol. 2023;79:433–460. 10.1016/j.jhep.2023.05.001 37364791

[16] Yurdaydin C Keskin O Kalkan Ç Karakaya F Çalişkan A Kabaçam G . Interferon treatment duration in patients with chronic delta hepatitis and its effect on the natural course of the disease. J Infect Dis. 2018;217:1184–1192. 10.1093/infdis/jix656 29425306

[17] Roulot D Brichler S Layese R BenAbdesselam Z Zoulim F Thibault V . Origin, HDV genotype and persistent viremia determine outcome and treatment response in patients with chronic hepatitis delta. J Hepatol. 2020;73:1046–1062. 10.1016/j.jhep.2020.06.038 32634548

[18] Mangia A Squillante MM Fraticelli F Cavorsi MC Paroni G Zaffarano L . HDV RNA levels and progression of hepatitis delta infection: A 14 year follow up experience in Italy. Cells. 2023;12:1413. 10.3390/cells12101413 PMC10217045 37408247

[19] Wedemeyer H Schöneweis K Bogomolov P Blank A Voronkova N Stepanova T . Safety and efficacy of bulevirtide in combination with tenofovir disoproxil fumarate in patients with hepatitis B virus and hepatitis D virus coinfection (MYR202): A multicentre, randomised, parallel-group, open-label, phase 2 trial. Lancet Infect Dis. 2023;23:117–129. 10.1016/S1473-3099(22)00318-8 36113537

[20] Anolli MP Degasperi E Allweiss L Sangiovanni A Maggioni M Scholtes C . A 3-year course of bulevirtide monotherapy may cure hdv infection in cirrhotics. J Hepatol. 2023;78:876–880. 10.1016/j.jhep.2022.12.023 36931396

[21] Degasperi E Anolli MP Jachs M Reiberger T De Ledinghen V Metivier S . Real-world effectiveness and safety of bulevirtide monotherapy for up to 96 weeks in patients with HDV-related cirrhosis. J Hepatol. 2025;82:1012–1022. 10.1016/j.jhep.2024.12.044 39793613

[22] Li D He W Liu X Zheng S Qi Y Li H . A potent human neutralizing antibody Fc-dependently reduces established HBV infections. Elife. 2017;6:e26738. 10.7554/eLife.26738 PMC5614562 28949917

[23] He W Ren B Mao F Jing Z Li Y Liu Y . Hepatitis D virus infection of mice expressing human sodium taurocholate co-transporting polypeptide. PLoS Pathog. 2015;11:e1004840. 10.1371/journal.ppat.1004840 PMC4406467 25902143

[24] He W Cao Z Mao F Ren B Li Y Li D . Modification of three amino acids in sodium taurocholate cotransporting polypeptide renders mice susceptible to infection with hepatitis D virus in vivo. J Virol. 2016;90:8866–8874. 10.1128/JVI.00901-16 PMC5021397 27466423

[25] Testoni B Lebossé F Scholtes C Berby F Miaglia C Subic M . Serum hepatitis B core-related antigen (HBcrAg) correlates with covalently closed circular DNA transcriptional activity in chronic hepatitis B patients. J Hepatol. 2019;70:615–625. 10.1016/j.jhep.2018.11.030 30529504

[26] Yan R Cai D Ouyang L Colonno R Huang Q Kitrinos KM . Development of a sensitive, multi-assay platform to monitor low levels of HBV DNA and pgRNA in patients with chronic hepatitis B virus infection. J Virol Methods. 2023;311:114640. 10.1016/j.jviromet.2022.114640 36332714

[27] Liu Y May L Liu X Martin R Svarovskaia E Gaggar A . Developing a sensitive HBV genotyping assay for HBV DNA suppressed patients using both DNA and RNA sequencing. J Med Virol. 2020;92:3420–3425. 10.1002/jmv.26249 32609910

[28] Sy BT Ratsch BA Toan NL Song LH Wollboldt C Bryniok A . High prevalence and significance of hepatitis D virus infection among treatment-naive HBsAg-positive patients in Northern Vietnam. PLoS One. 2013;8:e78094. 10.1371/journal.pone.0078094 PMC3799775 24205106

[29] Ghany MG Buti M Lampertico P Lee HM Faculty A-EH-HTEC . Guidance on treatment endpoints and study design for clinical trials aiming to achieve cure in chronic hepatitis B and D: Report from the 2022 AASLD-EASL HBV-HDV Treatment Endpoints Conference. Hepatology. 2023;78:1654–1673. 10.1097/HEP.0000000000000431 PMC12373085 37326326

[30] U.S. Food and Drug Administration. *Chronic Hepatitis D Virus Infection:* *Developing Drugs for Treatment Guidance for Industry (Draft guidance)*. U.S. Department of Health and Human Services. 2019.

[31] Blanchet M Sureau C . Infectivity determinants of the hepatitis B virus pre-S domain are confined to the N-terminal 75 amino acid residues. J Virol. 2007;81:5841–5849. 10.1128/JVI.00096-07 PMC1900317 17376925

[32] Sureau C Negro F . The hepatitis delta virus: Replication and pathogenesis. J Hepatol. 2016;64(1 suppl):S102–S116. 10.1016/j.jhep.2016.02.013 27084031

[33] Lampertico P Anolli MP Steppich K Wedemeyer H . Bulevirtide monotherapy or in combination for chronic hepatitis delta: 2025 update. J Viral Hepat. 2025;32:e70056. 10.1111/jvh.70056 PMC12644311 41287135

[34] Soriano V Moreno-Torres V Trevino A Corral O de Mendoza C . Bulevirtide in the treatment of hepatitis delta: Drug discovery, clinical development and place in therapy. Drug Des Devel Ther. 2023;17:155–166. 10.2147/DDDT.S379964 PMC9875571 36712949

[35] Bogomolov P Alexandrov A Voronkova N Macievich M Kokina K Petrachenkova M . Treatment of chronic hepatitis D with the entry inhibitor myrcludex B: First results of a phase Ib/IIa study. J Hepatol. 2016;65:490–498. 10.1016/j.jhep.2016.04.016 27132170

[36] Wedemeyer H Aleman S Blank A Andreone P Bogomolov P Chulanov V . 144 Weeks of bulevirtide monotherapy for chronic hepatitis D: Final and posttreatment results from a Phase 3 randomized trial. Gut. 2026;S0168-8278(29):00201-1 10.1016/j.jhep.2026.03.046 41966308

[37] Lampertico P Invernizzi F Viganò M Loglio A Mangia G Facchetti F . The long-term benefits of nucleos(t)ide analogs in compensated HBV cirrhotic patients with no or small esophageal varices: A 12-year prospective cohort study. J Hepatol. 2015;63:1118–1125. 10.1016/j.jhep.2015.06.006 26100495

[38] Pan C Gish R Jacobson IM Hu KQ Wedemeyer H Martin P . Diagnosis and management of hepatitis delta virus infection. Dig Dis Sci. 2023;68:3237–3248. 10.1007/s10620-023-07960-y PMC10374831 37338616

[39] Degasperi E Scholtes C Testoni B Renteria SU Anolli MP Charre C . Differential HBV RNA and HBcrAg patterns in untreated patients with chronic hepatitis delta. J Hepatol. 2025;82:1004–1011. 10.1016/j.jhep.2024.11.051 39662705

[40] Lucifora J Alfaiate D Pons C Michelet M Ramirez R Fusil F . Hepatitis D virus interferes with hepatitis B virus RNA production via interferon-dependent and -independent mechanisms. J Hepatol. 2023;78:958–970. 10.1016/j.jhep.2023.01.005 36702177

[41] Da BL Heller T Koh C . Hepatitis D infection: From initial discovery to current investigational therapies. Gastroenterol Rep (Oxf). 2019;7:231–245. 10.1093/gastro/goz023 PMC6691517 32477569

[42] Sagnelli E Felaco FM Petruzziello A Annella T Pasauale G Filippini P . Interaction between HDV and HBV infection in HBsAg-chronic carriers. Infection. 1991;19:155–158. 10.1007/BF01643238 1889868

[43] Meier MA Calabrese D Suslov A Terracciano LM Heim MH Wieland S . Ubiquitous expression of HBsAg from integrated HBV DNA in patients with low viral load. J Hepatol. 2021;75:840–847. 10.1016/j.jhep.2021.04.051 34004216

[44] Wooddell CI Yuen MF Chan HLY Gish RG Locarnini SA Chavez D . RNAi-based treatment of chronically infected patients and chimpanzees reveals that integrated hepatitis B virus DNA is a source of HBsAg. Sci Transl Med. 2017;9:eaan0241. 10.1126/scitranslmed.aan0241 PMC5830187 28954926

[45] Volz T Allweiss L ḾBarek MB Warlich M Lohse AW Pollok JM . The entry inhibitor Myrcludex-B efficiently blocks intrahepatic virus spreading in humanized mice previously infected with hepatitis B virus. J Hepatol. 2013;58:861–867. 10.1016/j.jhep.2012.12.008 23246506

[46] Allweiss L Volmari A Suri V Wallin JJ Flaherty JF Manuilov D . Blocking viral entry with bulevirtide reduces the number of HDV-infected hepatocytes in human liver biopsies. J Hepatol. 2024;80:882–891. 10.1016/j.jhep.2024.01.035 38340811

[47] Wedemeyer H Aleman S Brunetto MR Blank A Andreone P Bogomolov P . A phase 3, randomized trial of bulevirtide in chronic hepatitis D. N Engl J Med. 2023;389:22–32. 10.1056/NEJMoa2213429 37345876

