# Supplementary material for: Safety and efficacy of libevitug in patients with chronic hepatitis B and D virus coinfection: A single-center, open-label, phase IIa trial in China
Source: Hepatol Commun. 2026 Jul 15;10(8):e00974. doi: 10.1097/HC9.0000000000000974 (PMC13671617; doi:10.1097/HC9.0000000000000974)
Supplement: Supplementary file 1 [file hc9-10-e00974-s001.docx]

**Supplemental Digital Content**

**Safety and Efficacy of Libevitug in Patients with Chronic Hepatitis B and D Virus Co-Infection: A Single-Center, Open-Label, Phase IIa Trial in China**

First author: Xinrui Wang^1*^, Xiumei Chi^1*^

^1^ Department of Hepatology, Center for Pathogen Biology and Infectious Diseases, The First Hospital of Jilin University, Changchun, China

^*^ These authors contributed equally to this work

Supplementary methods

## Inclusion and exclusion criteria of the clinical trial

**Inclusion criteria**

1) Signed informed consent form (ICF);
2) Male or female subjects aged from 18 to 70 years inclusively;
3) 18 kg/m^2^≤BMI≤32 kg/m^2^, body weight ≥45 kg for men and ≥40 kg for women;
4) Chronic hepatitis B, characterized by persistent of HBsAg positivity for at least 6 months prior to screening, with concurrent hepatitis D virus co-infection, as evidenced by positive anti-HDV antibodies and HDV RNA at the screening visit;
5) Women of childbearing potential or male subjects with female partners of childbearing potential should agree to use an adequate and highly effective contraception from screening to the end of study or until 12 weeks after last dose of the study drug (whichever is longer);

**Exclusion criteria**

1) Be pregnant or lactating at screening;
2) Positive for Treponema pallidum antibodies with a positive rapid plasma reagin (RPR) test, or positive for human immunodeficiency virus (HIV) antibodies;
3) Decompensated cirrhosis (complications of portal hypertension and/or hepatic insufficiency based on cirrhosis), with cirrhosis diagnosis based on, but not limited to: liver imaging (e.g., ultrasound) or histopathological confirmation of cirrhosis within 6 months prior to screening (including screening period); or liver stiffness measurement (LSM) ≥12 kPa (for normal alanine aminotransferase [ALT]) or ≥17 kPa (for elevated ALT) at screening, with the more severe result taken as reference;
4) Hepatic insufficiency (including, but not limited to, ascites, hepatic encephalopathy, upper gastrointestinal bleeding) within 3 months prior to screening;
5) Persistent alcohol consumption (average daily intake >40 g for men, >20 g for women) or addiction to illicit drugs within 6 months prior to screening;
6) Coexisting severe systemic diseases deemed unsuitable for study participation by the investigator or sponsor, including but not limited to severe cardiac or pulmonary conditions, chronic or recurrent urinary tract diseases, uncontrolled diabetes, autoimmune disorders, or epilepsy requiring treatment;
7) History of hepatocellular carcinoma (HCC), or histopathological or radiological evidence suggestive of HCC;
8) History of non-HCC malignancies, unless the malignancy has been in complete remission for at least 3 years prior to screening without requiring chemotherapy or additional medical/surgical intervention;
9) Poorly controlled or unstable hypertension at screening (persistent systolic blood pressure ≥160 mmHg or diastolic blood pressure >95 mmHg), though subjects with well-controlled blood pressure prior to dosing may be included after discussion between the investigator and sponsor;
10) Clinically significant electrocardiogram (ECG) abnormalities, including but not limited to torsades de pointes, paroxysmal ventricular tachycardia, symptomatic atrial fibrillation/flutter requiring urgent treatment, or complete atrioventricular block;
11) History of allergy or hypersensitivity reactions to any component or excipient of the study drug or antibody-based therapies;
12) Any of the following laboratory abnormalities at screening:
    a) Platelet count <80,000/mm³;
    b) Albumin <35 g/L;
    c) Total bilirubin (TBil) >1.5× upper limit of normal (ULN), except in subjects with Gilbert syndrome;
    d) Direct bilirubin (DBil) >1.5×ULN;
    e) ALT >5×ULN;
    f) Serum alpha-fetoprotein (AFP) ≥100 ng/mL (or if AFP >50 ng/mL at screening, imaging [liver ultrasound and/or magnetic resonance imaging] reveals liver nodules >5 mm and HCC cannot be ruled out after consultation);
    g) International normalized ratio (INR) >1.3;
    h) Estimated glomerular filtration rate (eGFR) <60 mL/min/1.73 m², calculated using the Chronic Kidney Disease Epidemiology Collaboration (CKD-EPI) equation;
    i) Any other laboratory abnormality deemed clinically significant and unsuitable for study participation by the sponsor or investigator;
13) Interferon antiviral therapy within 1 year prior to screening.

## Rationale for dose selection

The clinical dose regimen of 20 mg/kg Libevitug administered intravenously every two weeks (Q2W) in this HH003-201 study was selected mainly based on the integrated pharmacokinetic, safety, and antiviral data from a phase Ib study (NCT05542979) in treatment naïve patients with HBeAg-positive chronic HBV infection.^[1]^ In the phase Ib study, participants received Libevitug intravenously Q2W at doses of 3, 10, 20, and 40 mg/kg for a total of three doses. The study established a favorable safety profile and a dose-dependent pharmacokinetic relationship. Among the doses tested, 20 mg/kg of Libevitug resulted in optimum dose exposure and antiviral activity in the chronic hepatitis B (CHB) participants, thereby justifying its selection for subsequent clinical development.

## HBV and HDV Genotyping

HBV genotypes and HDV genotypes were determined by sequence analysis based on Nested-PCR as previously described.^[2,3]^ In brief, total nuclear acid (TNA) was extracted from the serum samples of participants using the Maxwell RSC Viral Total Nucleic Acid Purification Kit (AS1330, Promega) on the Maxwell RSC 48 instrument (AS5800, Promega). For HBV genotyping, total nuclear acid was reverse transcribed using SuperScript™ IV VILO™ Master Mix (11756050, Thermo Fisher Scientific) with HBV specific primers HBPr135. Subsequently, both the synthesized cDNA and TNA were subjected to a two-step, nested PCR to amplify the S region, where HBPr135 and HBPr134, HBPr75 and HBPr94 served as outer primers and inner primers respectively. PCR products were purified with the SanPrep Column DNA Gel Extraction Kit (B518131, Sangon Biotech, China) and sequenced by Sanger sequencing. For HDV genotyping, total nuclear acid was reverse transcribed to cDNA and PCR using SuperScript^TM^ IV One-Step RT-PCR System (12594025, Thermo Fisher Scientific) with HDV specific primers (the outer sense and antisense primer pairs) followed by a nested PCR amplification of HDAg using Platinum^TM^ II Taq Hot-Start DNA Polymerase (14966001, Thermo Fisher Scientific) with the inner primer pairs. PCR products were purified and subjected to Sanger sequencing analysis. The primers utilized in HBV and HDV genotyping are listed in Supplementary Table 1. For samples in which HBV genotype could not be determined by sequencing, an enzyme immunoassay (EIA)-based HBV genotyping kit (Cosmo Bio, Japan) was employed to determine the genotype.

Phylogenetic analysis was performed to determine the genotypes of HBV and HDV using neighbor-joining method in MEGA11. HBV genotypes were established by comparison of the sequences obtained with the HBV S region sequences retrieved from GenBank, which includes the predominant global HBV genotypes (A, B, C, and D). The representative reference sequences utilized were as follows: HBV genotype A (LC519823.1), genotype B (LC456127.2), genotype C (LC535950.1), and genotype D (HE815465.1). HDV genotypes were established by comparison of the sequences obtained with the HDAg sequences retrieved from GenBank, which includes the known HDV genotypes 1-8. The reference sequences comprised: HDV genotype 1 (X77627.1, M92448.1, AB118848.1, NC001653, AJ000558, X85253, AY633627, AF098261); genotype 2 (AJ309880, X60193, U19598.1); genotype 3 (AB037948, AB037949, AB037947); genotype 4 (AF309420, AF018077, AB118847, AF209859); genotype 5 (AM13326.1, AX741154.1, AX741159.1, AX741149.1); genotype 6 (AM183332.1, AM183329.1); genotype 7 (AM183333.1, AJ584844.1); and genotype 8 (AM183330.1, AM183327.1, AX741169.1).^[4]^

## Drug resistance analysis

Drug resistance testing was conducted for participants meeting the following criteria: a confirmed increase in HBV DNA or HDV RNA levels of >1 log_10_ IU/mL from the nadir in two consecutive tests using the same method, with an interval of more than one month, in participants with prior at least >1 log_10_ IU/mL reduction from the baseline. Alternatively, a confirmed increase in HBV DNA or HDV RNA levels of >1 log_10_ IU/mL from the lower limit of quantification (LLOQ) or limit of detection (LOD) in those who previously achieved <LLOQ or LOD.

Genotypic drug resistance testing was performed using Sanger sequencing of the Libevitug-targeted HBV PreS1 region, the HBV reverse transcription (RT) region, and the HDAg region, at baseline and at the time of virological breakthrough, for participants who met the criteria outlined above. Total viral nucleic acids (RNA and DNA) were extracted from participant serum samples, followed by cDNA synthesis, PCR amplification, and Sanger sequencing analysis. The resulting sequences of the targeted regions were then aligned with corresponding baseline samples or with the reference HBV or HDV sequence database when baseline sequences were unavailable due to methodological limitations. The primers utilized in HBV and HDV genotypic drug resistance analysis are listed in Supplementary Table 1.

Supplement TABLE 1 Primers for hepatitis D virus/hepatitis B virus genotyping and genotypic drug resistance analysis

| **Primer name** | **Sequence (5'‐3')** | **Description** | **Application** |
| --- | --- | --- | --- |
| HBPr135 | CARAGACAAAAGAAAATTGG | Reverse transcription primer, outer antisense primer | HBV genotyping |
| HBVPr134 | TGCTGCTATGCCTCATCTTC | Outer sense primer |  |
| HBPr75 | CAAGGTATGTTGCCCGTTTGTCC | Nested sense primer |  |
| HBPr94 | GGYAWAAAGGGACTCAMGATG | Nested antisense primer |  |
| Outer sense primer | GCCCAGGTCGGACCGCGAGGA | One-Step RT-PCR outer sense primer | HDV genotyping |
| Outer antisense primer | ACAAGGAGAGGCAGGATCACCGAC | One-Step RT-PCR outer antisense primer |  |
| Inner sense primer | GAGATGCCATGCCGACCCGAAGAG | Nested sense primer |  |
| Inner antisense primer | GAGATGCCATGCCGACCCGAAGAG | Nested antisense primer |  |
| FA2-L | GGGTCGCAGAAGATCTCAAT | HBV PreS1 region sense primer | Genotypic drug resistance analysis of HBV PreS1 |
| HBV-367R | ACCAGGACAARTTGGAGGAC | HBV PreS1 region antisense primer |  |
| FA3-L | GACTCGTGGTGGACTTCTCTCAA | HBV RT region sense primer | Genotypic drug resistance analysis of HBV RT |
| HBVPr-1008R | AGACCCACAATTCKYTGACATAC | Reverse transcription primer, HBV RT region antisense primer |  |
| HDV-1631R | GAGWWCTSAGGACCGTCGCC | Reverse transcription primer | Genotypic drug resistance analysis of HDAg |
| HDV-860F | GCCCAGGTCGGACCGCGAGGA | HDAg region sense primer |  |
| HDV-1607R | TCGAAGATGAGCCRRYCCGAWKSRA | HDAg region antisense primer |  |

Abbreviations: HDAg, hepatitis delta antigen; RT, reverse transcription.

Supplementary TABLE 2 Genotypic drug resistance analysis results for participants with virological breakthrough

| **Participant ID** | **Amino acid change from baseline** | | |
| --- | --- | --- | --- |
|  | **HBV PreS1 region** | **HBV RT region** | **HDAg region** |
| Participant 03 | A22T^a^ | No variants^b^ | No variants |
| Participant 05 | No variants | No variants | No variants |
| Participant 07 | A22T/A | No variants | No variants |
| Participant 09 | F23L/F | No variants^b^ | No variants |
| Participant 10 | A22D/G/A | No variants | No variants |

Participants 03, 07, 09 experienced HBV virological breakthroughs only, while participant 05 and 10 exhibited both HBV and HDV virological breakthroughs. Participants (05, 07, 09, 10) experienced virological breakthroughs after missing a dose of Libevitug treatment due to COVID-19.

a: HBV PreS1 region sequences were not obtained at baseline due to low HBV DNA titers; virological breakthrough sequences were aligned with reference database sequences (https://hbvdb.lyon.inserm.fr).

b: HBV RT region sequences were not obtained at baseline due to low HBV DNA titers; virological breakthrough sequences were aligned with reference database sequences [(https://hbvdb.lyon.inserm.fr).](../../(https:/hbvdb.lyon.inserm.fr).)

Abbreviations: HBV, hepatitis B virus; HDAg, hepatitis delta antigen; RT, reverse transcription.

**Supplementary Figure 1 Viral genotypes analysis of participants enrolled in HH003-201 study.** Phylogenetic trees were reconstructed using neighbor-joining algorithm by comparing pairwise distances between nucleotide sequences of HBV S (s101-s213) coding region (A) or HDAg coding region (B) in MEGA11. The HBV genotypes of participants 10 and 17 were determined using an enzyme immunoassay (EIA)-based HBV genotyping kit (Cosmo Bio, Japan) instead of Sanger sequencing due to viral loads below the assay’s limit of detection. The HDV genotype of participant 09 was not obtained due to viral loads below the assay’s limit of detection and is absent from the phylogenetic tree.

**Supplementary Figure 2 Hepatitis B core-related antigen (HBcrAg) changes during treatment and follow-up period.** Individual, color-coded participant responses during the 24-week treatment and the 24-week follow-up are shown. Dashed gray lines indicate the LLOQ (3 log_10_ U/mL) for HBcrAg. Due to missed visits and (or) dosing, missing data were occurred for the following participants: participant 01 (week 32), participant 02 (week 32), participant 03 (week 32), participant 05 (week 20), participant 06 (week 12), participant 07 (week 12), participant 09 (week 8), participant 10 (week 12), and participant 17 (week 6). Abbreviations: HBcrAg, hepatitis B core-related antigen; LLOQ, lower limit of quantification.

**Supplementary Figure 3 Individual virological biomarkers and ALT profiles over time.** Individual HDV RNA, HBV DNA and ALT change kinetics during the study are shown. Missing dosing and visits are indicated by a red x, while the initiation of NrtI therapy is denoted by a blue star. Abbreviations: ALT, alanine aminotransferase; HBV, Hepatitis B virus; HDV, Hepatitis D virus.

**Supplementary Figure 4** **Virological biomarkers and ALT profiles over time for participants experienced ALT flares.** Individual HDV RNA, HBV DNA, HBsAg, HBcrAg, and ALT change kinetics for participant 06 (A) and participant 05 (B) who occurred ALT flare (defined as a greater than 3-fold increase in serum ALT level above baseline) during the study are shown. Missing dosing and visits are indicated by red x. Abbreviations: ALT, alanine aminotransferase; HBcrAg, hepatitis B core-related antigen; HBsAg, hepatitis B surface antigen; HBV, Hepatitis B virus; HDV, Hepatitis D virus.

**REFERENCE**

1.	Hu Y, Huang Y, Li G, et al. al. A first-in-patient phase Ib study of a hepatitis B virus (HBV) neutralizing antibody HH-003 in treatment naive participants with HbeAg-positive chronic HBV infection. AASLD; 2022.

2.	Liu Y, May L, Liu X, et al. Developing a sensitive HBV genotyping assay for HBV DNA suppressed patients using both DNA and RNA sequencing. *J Med Virol*. Dec 2020;92(12):3420-3425.

3.	Sy BT, Ratsch BA, Toan NL, et al. High prevalence and significance of hepatitis D virus infection among treatment-naive HBsAg-positive patients in Northern Vietnam. *PLoS One*. 2013;8(10):e78094.

4.	Joseph K, Shabangu CS, Jang TY, et al. The Prevalence and Serological Association of Hepatitis D Virus Genotypes in Taiwan. *Pathogens*. Sep 23 2021;10(10)
